# *Callicarpa dichotoma* Leaf Extract Alleviates Atopic Dermatitis through the Suppression of T Cells and Keratinocytes Activation

**DOI:** 10.3390/ph15101280

**Published:** 2022-10-18

**Authors:** Eun-Nam Kim, Hyun-Su Lee, Gil-Saeng Jeong

**Affiliations:** 1 College of Pharmacy, Chungnam National University, Daejeon 34134, Korea; 2 Department of Physiology, Daegu Catholic University School of Medicine, Daegu 42472, Korea

**Keywords:** atopic dermatitis, *Callicarpa dichotoma*, T cells, keratinocytes, interleukin, thymic stromal lymphopoietin

## Abstract

Atopic dermatitis (AD) is a highly recurrent chronic inflammatory skin disease, characterized by severe itching, immune imbalance, and skin barrier dysfunction. Damage to the skin barrier function is known to be the main cause of Th1/Th2 immune imbalance, due to the Th2-mediated immune response, and pro-inflammatory cytokines, including IL-4, IL-5, IL-13 and IL-31 and it plays an important role in further eliciting the environment of AD through stimulation. Currently, the most widely used drugs for the treatment of AD are corticosteroids, antihistamines and immunosuppressants (used by more than 60% of patients), which are reported to exhibit various side effects when taken for a long time. Therefore, interest in the physiological activity of safer plant-derived natural extracts is increasing. *Callicarpa dichotoma* is traditionally used in oriental medicine for bruises, habitual pain, gastric and postpartum hemorrhage. Recent studies have reported that it exhibits antioxidant anti-inflammatory and anti-hepatotoxic activity, but the role and activity of *C. dichotoma* in AD have not yet been studied. Therefore, in this study, the new physiological activity of *C. dichotoma* in the AD environment was investigated, suggesting its potential as a natural therapeutic agent.

## 1. Introduction

Atopic dermatitis (AD) is a chronic inflammatory skin disease accompanied by severe itching and it is known worldwide as a disease in both children and adults that is caused by immune imbalance and skin barrier dysfunction. [1,2]. AD is a representative T-cell immune-mediated disease and is characterized by several factors, such as skin redness, dryness, itching, keratinocyte proliferation, immune cell infiltration, and lymph node T-cell expansion. In particular, Th1/Th2 immune imbalance caused by Th2-mediated immune response causes AD, along with damage to the skin barrier function [3]. Immune response by immunologically abnormal Th2 cells is induced by high antibody production, and plays a role in activating B cell proliferation and class conversion, thereby causing cytokines interleukin (IL)-25, IL-33 and thymic stromal lymphopoietin (TSLP) to increase in keratinocytes of AD patients [4,5,6]. Cytokines IL-25, IL-33, and TSLP play an important key role in the pro-inflammatory milieu of AD by stimulating Th2 cells directly or indirectly through the stimulation of multiple immune cells, including dendritic cells, mast cells, and eosinophils, and immunomodulatory disorders and contribute to barrier dysfunction [7,8]. These processes can also activate the cytokines IL-4, IL-5, IL-13 and IL-31, which are known to play a major role in skin infiltration in AD skin lesions [9,10,11]. Currently, the most widely used drugs for the treatment of AD include corticosteroids, antihistamines, and immunosuppressive drugs, which are used by more than 60% of patients [12,13]. However, the use of these drugs to relieve symptoms of AD leads to various tolerance and side effects to the drug, including glaucoma, impaired wound healing, and growth retardation [14]. Therefore, in order to reduce these problems, therapeutic agents that can replace the existing drugs are being developed to obtain safer and more effective therapeutics, and interest in the physiological activity of natural extracts derived from safer plants has started to increase recently [15,16].

*Callicarpa dichotoma* (Verbenaceae) is distributed evenly across Korea and central Japan, and it is known that there are about 40 species of plants that belong to *Callicarpa* worldwide [17]. In oriental traditional medicine, the plant of the genus *Callicarpa* has been used for bruises, habitual pain, gastric and postpartum hemorrhage, and the leaves in particular have been used to treat inflammation and gastric ulcers [18,19]. The leaves and twigs of *C. dichotoma* contain various types of phenylethanoid glycosides, essential oils, diterpenes, and flavonoids [20]. In a previous study, the compound acteoside, isolated from *C. dichotoma,* reduced neurotoxicity caused by glutamate and was reported to exhibit antioxidant anti-inflammatory and anti-hepatotoxic activity [20]. Research on the ingredients and activities *of C. dichotoma* is still incomplete. In addition, despite the anti-inflammatory effect of *C. dichotoma*, its application to various inflammatory diseases has not been attempted. Therefore, in this study, the activity of *C. dichotoma* was evaluated on an in vivo experimental animal model of mite-induced atopic dermatitis, and the effect of *C. dichotoma* on the activity of T cells and keratinocytes, known as the main cause of atopic dermatitis, was investigated.

## 2. Results

### 2.1. Major Ingredient Chemoprofile of CDE

**Figure 1:**
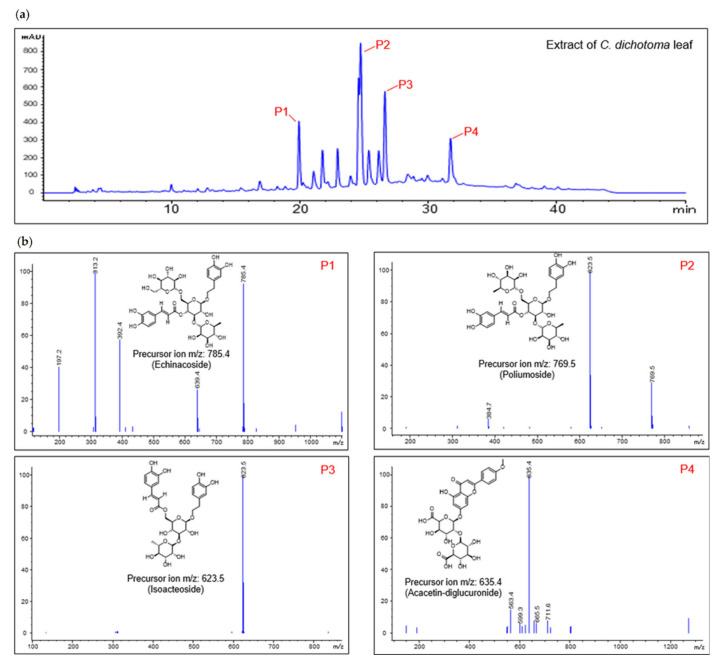
Major ingredient chemoprofile of CDE. (**a**) HPLC-MS chromatograms of CDE peaks. ESI-MS spectra of each compound precursor ion ([M-H]^−^ ion) (**b**).

Qualitative analysis was performed using HPLC-MS for the chemical profile analysis of CDE, and the molecular weights of the major four component peaks were compared with previously reported literature [21]. First, the molecular weight of the main component was confirmed from liquid chromatography (LC), as shown in Figure 1a. As a result, peak 1 (P1) was identified as echinacoside with a molecular weight of 786.4, peak 2 (P2) as poliumoside with a molecular weight of 770.7, peak 3 (P3) as isoacteoside with a molecular weight of 624.6, and peak 4 (P4) as acacetin-diglucuronide with a molecular weight of 636.5 (Figure 1b).

### 2.2. Oral Administration of C. dichotoma Leaf Extract (CDE) Suppresses Atopic Dermatitis in Mice Model

**Figure 2:**
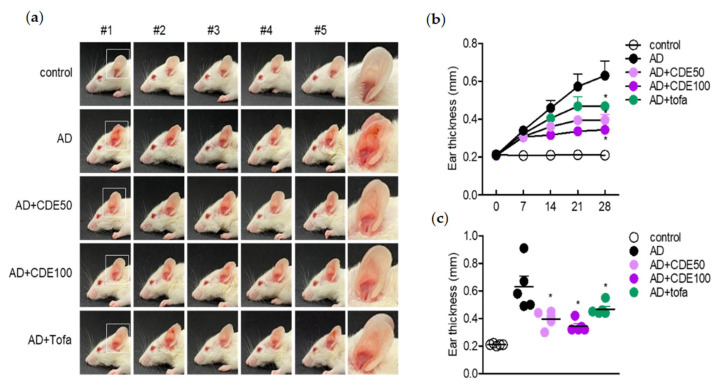
Oral administration of CDE suppresses atopic dermatitis in mice model. (**a**) Representative photograph of mice ears for each experimental group. (**b**) Individual ear thickness for each mouse at 14 and 28 days after AD induction. (**c**) Mean ear thickness for each group of mice at 14 and 28 days after AD induction. As a positive control group, 30 mg/kg of tofacitinib was administered. Results are presented as mean ± SEM of 5 mice (* *p* < 0.05).

To explore the AD treatment effect of CDE, we evaluated the effect of oral administration of CDE in AD-induced mice stimulated with DNCB/mite. As an evaluation item, it was checked whether the thickness of the ear tissue in the AD state and parakeratosis, when observed with the naked eye, were reduced by oral administration of CDE. The data confirmed that the thickness of the ear tissue increased in the AD induction group compared to the control group, and it was confirmed that the red parakeratosis was also reduced (Figure 2a). Then, it was asserted that the symptoms of AD were alleviated by oral administration of CDE, and the effects of the CDE 50 and 100 μg/mL oral administration groups were superior to the positive control of the tofacitinib-treated group (Figure 2b,c). These results suggest the potential of CDE as a modulator of AD by alleviating the typical symptoms of AD.

### 2.3. CDE Modulates the mRNA Levels of Pro-Inflammatory Cytokines on Ear Tissues

**Figure 3:**
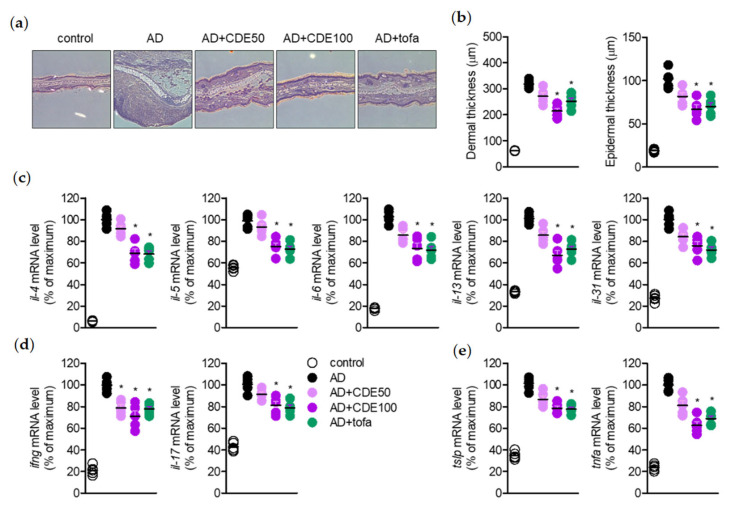
CDE modulates the mRNA levels of pro-inflammatory cytokines in ear tissues. (**a**) Representative histological stain images of mice ears (H&E staining). (**b**) Dermal and epidermal thickness of each mouse ear from histological stain images of mice ears. (**c**–**e**) The mRNA level of pro-inflammatory cytokines in ear tissues was detected by real-time quantitative PCR. The groups in the experiment were as follows: mock, mice group treated with PBS; AD, mice group with induced atopic dermatitis by application of mite extract and 1% DNCB; AD + CDE 50 mg/kg, mice group with induced atopic dermatitis by application of mite extract and 1% DNCB; AD + CDE 100 mg/kg, tofacitinib was used as a positive control. Results are expressed as mean ± SEM of five mice (* *p* < 0.05).

In addition, in order to support the hypothesis that CDE has an alleviating effect on AD symptoms, we evaluated the effect of CDE on the mRNA expression of major pro-inflammatory cytokines in AD from ear tissues. The immune response by Th2 cells induces the production of pro-inflammatory cytokines, such as IL-4, 5, 13, 31 and TSLP, and these pro-inflammatory cytokines cause the pro-inflammatory environment of AD through Th2 cell stimulation. Hence, the dermal and epidermal thickness of the ear tissue caused by AD was measured through H&E tissue staining of the ear tissue and the effect of CDE on the production of these pro-inflammatory cytokines was evaluated. The results showed that CDE stably recovered the dermal and epidermal thickness of the ear tissue that was thickened by AD induction (Figure 3a,b), and showed superior effects compared to tofacitinib, the positive control, and also down-regulated the gene expression of pro-inflammatory cytokines (Figure 3c–e). Therefore, it is believed that the AD-alleviating effect of CDE is due to the downregulation of these pro-inflammatory cytokines, and it can be said to be the evidence that can further support the potential of CDE as an AD reliever.

### 2.4. CDE Reduces IgE Production and Mast Cell Infiltration in AD Mice

**Figure 4:**
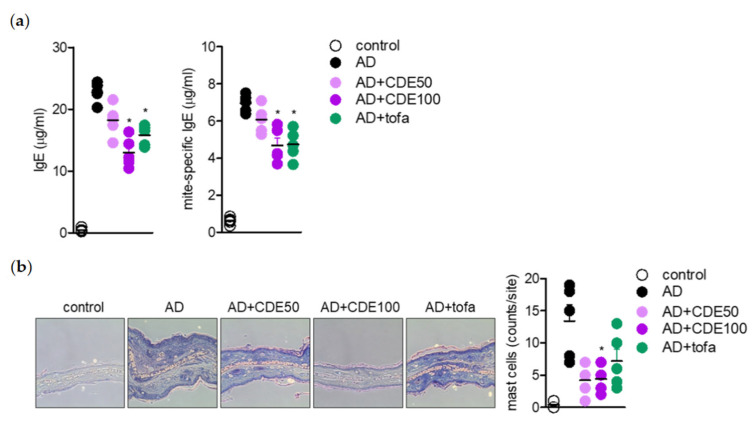
CDE reduces IgE production and mast cell infiltration in AD mice. (**a**) Levels of IgE and mite-specific IgE were analyzed by ELISA in serum isolated on day 28 after induction. (**b**) Representative photos of sections for each group through toluidine blue staining and evaluation of the number of mast cell infiltration into the ear tissues of each group. Results are expressed as mean ± SEM of five mice. * *p* < 0.05, versus the AD group.

As more convincing evidence for the AD regulating effect of CDE, the amount of immunoglobulin E (IgE), a representative marker in AD, and the number of mast cells capable of binding to IgE in AD-induced mice were evaluated. As a result, CDE down-regulated the levels of IgE and the mite-specific IgE was increased by AD induction. In particular, the CDE 100 μg/mL oral administration group showed a significant IgE down-regulation effect compared to the positive control group (Figure 4a). In addition, CDE down-regulated the number of mast cells, suggesting its potential to down-regulate mast cell binding potential to high-affinity IgE receptors (Figure 4b). These results suggest that CDE improves the treatment of disease through improving AD-related symptoms and gene regulation through the down-regulation of IgE and mite-specific IgE, as well as the increased dermal/epidermal thickness caused by AD and the down-regulation of pro-inflammatory cytokines.

### 2.5. CDE Blocks Systemic Immune Responses in Atopic Dermatitis

**Figure 5:**
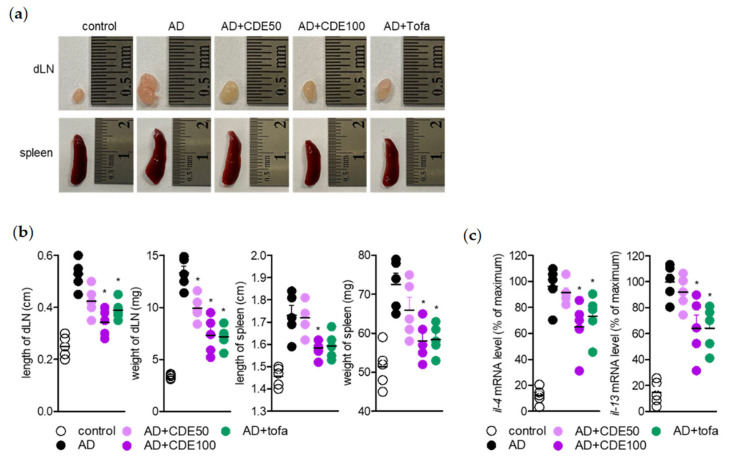
CDE blocks systemic immune responses in atopic dermatitis. (**a**) Representative photographs of draining lymph nodes and spleen in each group. (**b**) Length and weight of draining lymph nodes and spleen for each mouse at day 28 after AD induction. (**c**) mRNA levels of T cell-mediated cytokines in draining lymph nodes were detected by real-time quantitative PCR. Results are presented as mean ± SEM of 5 mice (* *p* < 0.05).

As shown in the figures, CDE is thought to alleviate the symptoms of AD through the downregulation of pro-inflammatory cytokines and IgE related to the immune response. Therefore, in order to confirm whether the AD mitigation effect of CDE occurs through systemic immune response regulation, the length and weight of draining lymph nodes and spleen were measured, which have an important effect on the response to T cell immunity during AD development. As a result, oral administration of CDE effectively reversed the effect on hypertrophic draining lymph nodes (dLN) and spleen length and weight caused by AD induction (Figure 5a,b). In addition, among the major cytokines in AD, Th2-related pro-inflammatory cytokines are produced in activated T cells of dLN, so the gene levels of related cytokines *il4* and *il13* were confirmed. As a result, CDE downregulated the gene levels of *il4* and *il13* that were increased by AD induction (Figure 5c). These results suggest that the AD mitigating effect of CDE can be achieved through the regulation of activated T cells, suggesting its potential as a modulator of the systemic immune response.

### 2.6. CDE Inhibits the Activity of T Cells without Cytotoxicity

**Figure 6:**
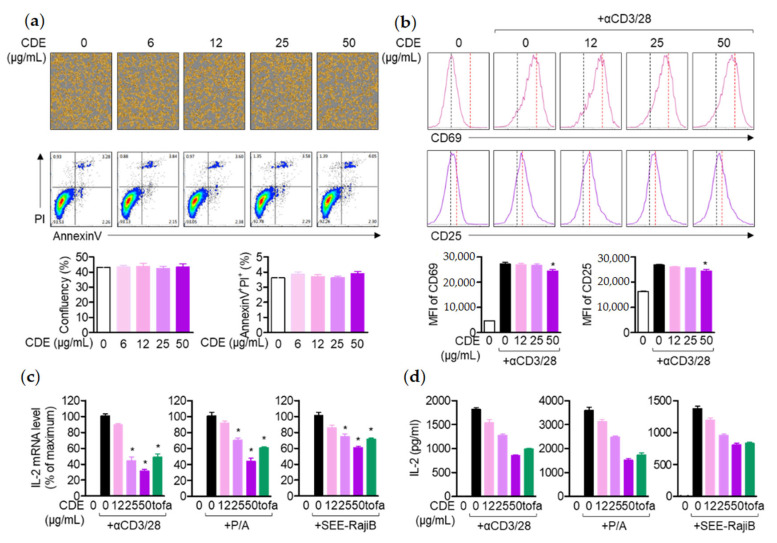
CDE inhibits the activity of T cells without cytotoxicity. (**a**) CDE did not show cytotoxicity; (**b**) Jurkat T cells were treated with CDE at the indicated concentrations for 1 h and then stimulated with anti-CD3 and CD28 antibodies for 6 h. Then, the harvested cells were dyed with anti-CD69 antibodies or APC connected with APC. The dyed cells were obtained by the cell analysis for fluorescence detection and the average fluorescent strength was obtained. The black dotted line shows the average fluorescence of the simulated cells, and the red dotted line indicates the average fluorescence of the anti-CD3/28 antibody. (**c**,**d**) In addition, Raji B cells (right) were loaded after stimulation with CD28 antibody, PMA (100 nM) and SEE (1 μg/mL). The effect of IL-2 on mRNA levels was analyzed by real-time quantitative PCR, and released IL-2 was detected in each supernatant by ELISA. Results are expressed as mean ± SEM of three independent experiments (* *p* < 0.05).

As in the previous in vivo results, we evaluated whether CDE induces apoptosis in Jurkat T cells in order to obtain more convincing evidence that CDE exhibits the symptom-relieving effect of AD through immunomodulation. Then, the effect of CDE was evaluated on IL-2 production in T cells-activated with anti-CD3/CD28 antibodies and PMA/A23187. In the evaluation results, it was revealed that CDE did not exert toxicity and it had no influence on the confluency of Jurkat T cells or apoptosis (Figure 6a). In addition, it was confirmed through flow cytometry that CDE downregulates the expression of upstream surface molecules in activated T cells, such as CD69 and CD25 in Jurkat T cells stimulated with the anti-CD3/CD28 antibody (Figure 6b). Therefore, to evaluate the effect on IL-2 generated from T cells activated by the anti-CD3/CD28 antibody and PMA/A23187, it was checked whether pretreatment with CDE inhibited IL-2 release from cells. As a result, CDE down-regulated the *il2* gene level released from activated T cells in a concentration-dependent manner (Figure 6c,d), suggesting that the AD mitigation effect of CDE on the immune response is achieved through the regulation of activated T cells.

### 2.7. The Activity of Keratinocytes Is Regulated by Pre-Treatment with CDE

**Figure 7:**
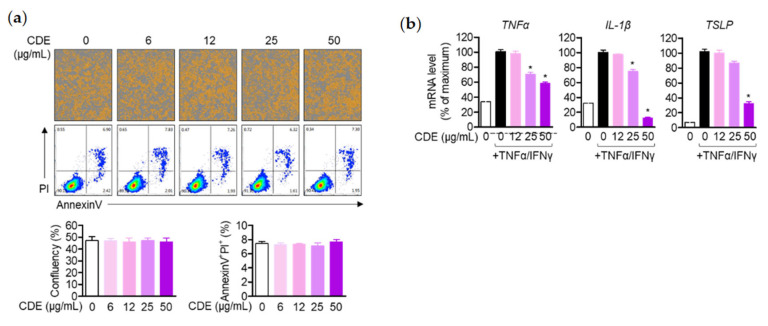
The activity of keratinocytes is regulated by pre-treatment with CDE. (**a**) The indicated concentrations of CDE (0–50 μg/mL) were incubated with Jurkat cells for 24 h, using a staining reagent for IncuCyte (1X AnnexinV) to detect AnnexinV, and the intensity of AnnexinV was obtained with the IncuCyte imaging system. For the determination of the AnnexinV + PI+ population, an AnnexinV/PI apoptosis assay was performed, using cells cultured according to the manufacturer’s instructions. (**b**) The mRNA levels of inflammatory cytokine genes were assessed by qPCR, and all gene expression was normalized to *gapdh* expression. Results are expressed as mean ± SEM of three independent experiments (* *p* < 0.05).

Based on previous in vitro results, we demonstrated the AD relief effect through systemic immune regulation of CDE; in addition, keratinocytes, representative cells that constitute the epidermis, were used to evaluate the effect of CDE on the production of several pro-inflammatory cytokines that contribute to the pathogenesis of AD. CDE did not show toxicity in keratinocytes or in Jurkat T cells in the indicated concentrations and did not affect the cellular confluency (Figure 7a). In addition, the increased pro-inflammatory cytokines TNF-α, IL-1β and TSLP in TNF-α/IFNγ-stimulated keratinocytes were downregulated in a concentration-dependent manner by CDE treatment (Figure 7b). Taking these results together, CDE not only regulates the systemic immune response, but also down-regulates the inflammatory response of keratinocytes that act as a protective barrier in the epidermis, thereby providing further evidence of AD alleviation and its potential as a therapeutic agent.

### 2.8. Modulatory Effects of CDE on the Activity of T Cells and Keratinocytes Involve the NFκB-MAPK Pathway

**Figure 8:**
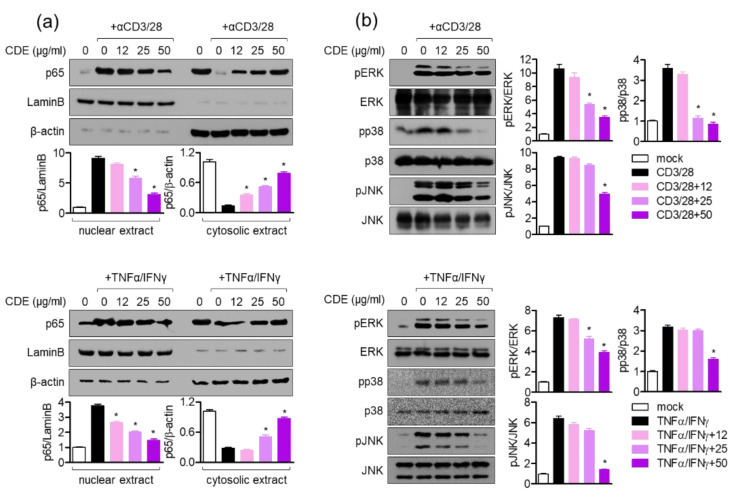
Modulatory effects of CDE on the activity of T cells and keratinocytes involve the NFκB-MAPK pathway. (**a**) Jurkat cells pretreated with the indicated concentrations of CDE (0–50 μg/mL) were stimulated with anti-CD3 antibody and anti-CD28 antibody for 10 min, then the cells were harvested and analyzed using Western blot analysis. (**b**) In addition, the indicated concentrations of CDE (0–50 μg/mL) were stimulated with anti-CD3 and anti-CD28 antibodies for 30 min after 1 h of treatment. Thereafter, the cells were lysed in RIPA buffer that contained 1X phosphatase inhibitor and then subjected to Western blot analysis to detect phosphorylated ERK, phosphorylated p38, and phosphorylated JNK. Phosphorylated levels were normalized to the intensity of total protein and displayed as bar graphs. Results are presented as the mean ± SEM of three independent experiments (* *p* < 0.05).

In order to explain how CDE has an enhancement effect on T cell activity during AD development, the effect of CDE on NFκB and MAPK was evaluated, which play important roles in the mechanism of the generation of pro-inflammatory cytokines in the activation process of AD through the Th2 immune response. First, CDE suppressed the increased translocation of NFκB p65 in Jurkat T cells and keratinocytes stimulated with the anti-CD3/CD28 antibody and TNFα/IFNγ, respectively, in a concentration-dependent manner (Figure 8a). In addition, it was confirmed that CDE also down-regulates phosphorylation levels of ERK, p38, and JNK (Figure 8b). These regulatory effects of CDE on NFκB nuclear translocation and MAPK phosphorylation are consistent with the previously reported basic mechanism of inhibitory effects on T cell activation via MAPK.

## 3. Discussion

The present study explored the mitigating effect of CDE on the symptoms and pathogenesis of AD by modulating T cells and keratinocytes. As a barrier against external environmental stimuli, the keratin of the skin contributes to ILC2 and Th2 cell activation and hypersensitivity reaction and, eventually, along with the activation of T cells, it induces the characteristics of AD [22]. Additionally, the immune response regulation through keratin protection from such damage to the skin barrier is reported as a new therapeutic strategy for AD [23]. Therefore, in this study, the effect of CDE on AD in this novel treatment strategy was demonstrated in vitro and in vivo. In this study, CDE effectively restored the thickened ear thickness of AD-induced mice, and also stabilized the thickness of the epidermis and dermis of the ear tissue. These results suggest that CDE alleviates the typical symptoms of AD and exhibits therapeutic effects. In this process, modulation of the immune response through keratin protection is known to be an important strategy for AD treatment [24]; therefore, the role of CDE in keratinocyte protection was also explored in this study.

AD is a representative Th2 cytokine gene expression-induced immune regulation disorder and various pro-inflammatory cytokines, in particular IL-4 and IL-13, have been studied as major therapeutic targets for AD [25,26]. In order to obtain more convincing evidence of the AD symptom-alleviation effect of CDE, the analysis of these main therapeutic target cytokines for AD was conducted from ear tissues of AD-induced mice. In addition, enlarged ear thickness in the AD-induced mouse model is a known symptom of AD [27], and the effect of CDE on these symptoms was evaluated together. CDE effectively down-regulated the gene expression levels of major pro-inflammatory cytokines, which are therapeutic targets for AD, and the ear thickness of the thickened mice was alleviated. Therefore, it could be concluded that CDE not only relieves typical symptoms of AD, but also exerts a therapeutic effect by directly down-regulating the therapeutic target cytokines.

Regarding the AD alleviation effect of CDE, as mentioned above, the effect of CDE on lymph nodes and spleen, which are representative immune organs, was evaluated to determine whether it is due to the effect of regulating the immune response through keratin protection. The lymph node, a representative primary immune organ, is known to play an important key role in the maturation of B cells and T cells, and the spleen, a secondary immune organ, is also known as another important institution [28,29]. According to the study results, CDE effectively restored the size of lymph nodes and spleen in mice with enlarged organs due to AD induction, which suggests that CDE can exert an AD-alleviating effect by modulating the systemic immune response. Therefore, as mentioned above, T cells activated in AD are known to induce the production of Th2 cytokines. This study assessed the direct effect of AD treatment of CDE on T cells and keratinocyte activities. As mentioned above, activated T cells in AD are known to induce the production of Th2 cytokines, and CDE effectively downregulated the expression of these cytokines. Therefore, in order to evaluate the direct effect of the AD treatment effect of CDE on the activity of T cells and keratinocytes, the expression levels of CD69 and CD25 were evaluated in Jurkat T cells stimulated with CD3/28. The *il2* gene expression level, a major cytokine of T cell activity, was evaluated. The data revealed that CDE did not show any effect on the toxicity of Jurkat T cells or keratinocytes in the indicated concentrations. Additionally, not only the expression levels of CD69 and CD25 of Jurkat T cells, but also the gene expression level of *il2* were down-regulated. Similarly, the gene expression levels of pro-inflammatory cytokines TNFα, IL-1β, and TSLP were suppressed in keratinocytes.

IL-4 is known to provide positive feedback to Th 2 cells and induce B isoform cells to differentiate into IgE antibody-producing cells, which is a major mediator and activator of allergic reactions, by inducing mitogen-activated protein kinase (MAPK) activation through the nuclear factor kappa-light-chain-enhancer of the activated B cells (NFκB) pathway to increase intracellular calcium and simultaneously induce histamine secretion [30,31]. In this study, CDE downregulated the *il4* gene expression level in AD-induced in vivo mice model; hence, the effect of CDE on the NFκB and MAPK pathways was evaluated. CDE inhibited the nuclear translocation of NFκB p65 in Jurkat T cells and keratinocytes stimulated with CD3/28 and TNFα/IFNγ, respectively, and downregulated the expression level of phosphorylated MAPK protein.

## 4. Materials and Methods

### 4.1. Plant Material

*C. dichotoma* was obtained at the Daegu Yangnyeong Herbal Medicine Market in 2021, and a *C. dichotoma* voucher specimen was deposited at Chungnam National University, College of Pharmacy.

### 4.2. Extract of C. dichotoma Leaf

The dried leaves of *C. dichotoma* (387.6 g) were manually separated and ground, and the dried leaves were extracted with EtOH (1 L) after 1 day at room temperature, and extracted twice for 2 h at 80 °C. Thereafter, the alcohol extract was concentrated in a rotary vacuum to obtain a residue (94.6 g).

### 4.3. Condition of Liquid Chromatography–Mass Spectrometry Analysis

To analyze the extract of *C. dichotoma* leaf, we used the single quadrupole negative ion mode on an eclipse plus C18 (5 μm × 4.6 mm × 250 mm; Agilent, Santa Clara, CA, USA) column and a high-performance liquid chromatography (Agilent model 1260 series, Santa Clara, CA, USA)–mass spectrometry (Agilent 6120 series, Santa Clara, CA, USA) system.

### 4.4. Reagents and Antibodies

Dinitrochlorobenzene (DNCB), and RIPA buffer were purchased from Sigma Chemical Co. (St. Louis, MO, USA). House dust mite (Dermatophagoides farina) extract was obtained from Greer (Lenoir, NC, USA). Mouse IgE ELISA kit was purchased from R&D Systems (Minneapolis, MN, USA). Antibodies for p65, p38, ERK, JNK, lamin B and β-actin were obtained from Cell Signaling Technology (Danvers, MA, USA). Antibodies for phosphorylated ERK, p38 and JNK were also purchased from Cell Signaling Technology (Danvers, MA, USA).

### 4.5. Animals

Six to eight-week-old female BALB/c mice were obtained from Samtako and housed in specific pathogen-free (SPF) conditions. All experiments were approved by the Animal Care and Use Committee of the College of Pharmacy, Keimyung University (approval number: KM2020-007, approved on July 2nd 2020).

### 4.6. Induction of Atopic Dermatitis

AD was induced by repeatedly applying mite extract and DNCB to the ears of mice. The control mice group were treated with DNCB/mite extract alone (AD), the experimental mice group were treated with DNCB/mite extract concurrently (mite extract and CDE (AD + CDE)), and for the positive control mice, DNCB/mite extract and tofacitinib (AD + tofa) were administered simultaneously. To induce AD disease, both ears were wrapped with surgical tape 5 times (Seo-il Chemistry, Hwasung, Korea). After stripping of the tape, 20 μL of DNCB (1%) was topically administered to each ear. After 4 days, the ears were painted with 20 μL of tick extract (10 mg/mL). Tick extract/DNCB treatment was repeated weekly for 4 weeks. Treatment started one day after the second DNCB application. It was repeated daily for 4 days thereafter. After a 2-day break, this 5-day on and 2-day off CDE dosing protocol was repeated for 4 weeks. Ear thickness was assessed 24 h after application of DNCB or mite extract using a dial thickness meter (Kori Seiki MFG Co., Tokyo, Japan). Animals were sacrificed 28 days after induction.

### 4.7. H&E Staining

H&E staining was performed to evaluate the histopathological effect of CDE on AD-induced mice. Ears removed from the experimental animals were fixed in 10% paraformaldehyde and embedded in paraffin. Paraffin-embedded ears were cut to a thickness of 5 μm, deparaffinized, and stained with hematoxylin and eosin (H&E). Dermal and epidermal thickness were measured on H&E-stained slides.

### 4.8. Cell Culture

Jurkat T cells (KCLB number: 40152) were purchased from the Korean Cell Line Bank (Seoul, Korea) and Raji B (ATCC cat#. CCL-86) cells were purchased from ATCC (Manassas, VA, USA). Cells were cultured in RPMI medium (Welgene, Gyeongsan, Korea), supplemented with penicillin G (100 units/mL), streptomycin (100 μg/mL), 10% fetal bovine serum (FBS), and L-glutamine (2 mM). In addition, the HaCaT cell line was kindly donated by Prof. Eun-Kyung Kim (Department of Food Science and Nutrition, Dong-A University, Busan, Korea) and cultured in DMEM medium (Welgene, Gyeongsan-si, Korea), supplemented with 10% fetal bovine serum (FBS), penicillin G (100 units/mL), streptomycin (100 μg/mL), and L-glutamine (2 mM). The passage of HaCaT cells was maintained at three to eight cells for experiments. Cells were grown at 37 °C in a humidified incubator that contained 5% CO_2_ and 95% air.

### 4.9. Analysis of mRNA Level Using Real-Time Quantitative PCR

To evaluate the mRNA level of each gene, cells were lysed in TRIZOL reagent. Then, reverse transcription of RNA was performed using the RT PreMix kit (Enzynomics, Korea). The primers used in this study were as follows: human *IL-2*, 5′-CAC GTC TTG CAC TTG TCA C-3′ and 5′-CCT TCT TGG GCA TGT AAA ACT-3′; human *tnfa*, 5′-ACC TCA TCT ACT CCC AGG TC-3′ and 5′-AAG ACC CCT CCC AGA TAG AT-3′; human *IL-1b*, 5′-GGA TAT GGA GCA ACA AGT GG-3′ and 5′-ATG TAC CAG TTG GGG AAC TG-3′; human *tslp*, 5′-TAG CAA TCG GCC ACA TTG CCT-3′ and 5′-GAA GCG ACG CCA CAA TCC TTG-3′; human *GAPDH*, 5′-CGG AGT CAA CGG ATT TGG TCG TAT-3′ and 5′-AGC CTT CTC CAT GGT GGT GAA GAC-3′; mouse *IL-4*, 5′-ACA GGA GAA GGG ACG CCA T-3′ and 5′-GAA GCC GTA CAG ACG AGC TCA-3′; mouse *Il-5*, 5′-GAA GTG TGG CGA GGA GAG AC-3′ and 5′-GCA CAG TTT TGT GGG GTT TT-3′; mouse *IL-6*, 5′-CCG GAG AGG AGA CTT CAC AG–3′ and 5′-GGA AAT TGG GGT AGG AAG GA–3′; mouse *IL-13*, 5′-GCA ACA TCA ACA GGA CCA GA–3′ and 5′-GTC AGG GAA TCC AGG GCT AC–3′; mouse *IL-31*, 5′- TCG GTC ATC ATA GCA CAT CTG GAG–3′ and 5′- GCACAG TCC CTT TGG AGT TAA GTC –3′; mouse *Ifng*, 5′-TCA AGT GGC ATA GAT GTG GAA GAA-3′ and 5′-TGG CTC TGC AGG ATT TTC ATG-3′; mouse *Il17*, 5′-TCC CCT CTG TCA TCT GGG AAG-3′ and 5′-CTC GAC CCT GAA AGT GAA GG-3′; mouse *TSLP*, 5′-GCA ACA TCA ACA GGA CCA GA–3′ and 5′-GTC AGG GAA TCC AGG GCT AC–3′; mouse *tnfa*, 5′-AAG CCT GTA GCC CAC GTC GTA-3′ and 5′-GGC ACC ACT AGT TGG TTG TCT TTG-3′; mouse *Gapdh*, 5′–GCA CAG TCA AGG CCG AGA AT–3′ and 5′–GCC TTC TCC ATG GTG GTG AA–3′. PCR amplification was performed in a DNA Engine Opticon 1 continuous fluorescence detection system (MJ Research, Waltham, MA, USA), using SYBR Premix Ex Taq. It contained 1 μL of cDNA/control and gene-specific primers. Each PCR reaction was performed using the following conditions: 95 °C 30 s, 60 °C 30 s, 72 °C 30 s, and plate reading (detection of fluorescent product) was carried out for 40 cycles, followed by 7 min of extension at 72 °C. Melting curve analysis was performed to characterize the dsDNA product by slowly raising the temperature (0.1 °C/s) from 60 °C to 95 °C, with fluorescence data collected at 0.2 °C intervals. mRNA levels of genes were normalized to Gapdh. The gene expression was calculated using the following equation: gene expression = 2−ΔΔCT, where ΔΔCT = (CT target−CT *gapdh*).

### 4.10. Analysis of Cell Confluency

Jurkat cells (1 × 10^4^ cells/well, 96 well) and keratinocytes (1 × 10^4^ cells/well, 96 well) were treated with the indicated concentration (6–50 μg/mL) of CDE for 24 h, then the cells were automatically marked in yellow by the IncuCyte live cell imaging system (Sartorius, Göttingen, Germany).

### 4.11. AnnexinV/PI Apoptosis Assay

To evaluate the effects of CDE on apoptosis, the AnnexinV/PI apoptosis kit was used. For determination of apoptosis after treatment with CDE, the AnnexinV/PI apoptosis kit was used. The Jurkat cells (5 × 10^5^/well, 12 well) and keratinocyte cells (5 × 10^5^/well, 12 well) were treated with CDE at the indicated concentrations (6–50 μg/mL) for 24 h and then stained with AnnexinV and PI, according to the manufacturer’s instructions. After one day, flow cytometry was performed and gated using BD software.

### 4.12. T Cell Stimulation

In this study, three methods were used to stimulate T cells. In the first method, Jurkat T cells were replaced on plates coated with anti-CD3 antibody (20 μg/mL) and stimulated by treatment with anti-CD28 soluble antibody (7 μg/mL). In addition, for PMA/A23187 stimulation, Jurkat T cells were stimulated by treatment with 100 nM PMA and 1 μM A23187, and as the final method, Jurkat T cells were stimulated with the same number of previously pulsed SEE superantigens (1 μg/mL) for 1 h and were stimulated by co-culture with Raji B cells.

### 4.13. Western Blot Analysis

Western blot analysis was performed to detect the level of each target protein in the cells. Lysates were obtained by lysing the harvested cells in RIPA buffer for 30 min on ice and centrifugation at 14,000 rpm for 20 min at 4 °C. For nuclear isolation of NFκB p65, NE-PER nuclear and cytoplasmic extraction reagents (Thermo Fisher Science, Waltham, MA, USA) were used according to the manufacturer’s instructions. Lysates (30–40 μg) were loaded for separation on an 8–12% SDS-PAGE gel. Total proteins were transferred to PVDF membranes (Bio-Rad, Hercules, CA, USA) and the membranes were placed in 5% skim milk in TBS that contained 0.1% Tween 20 (TBS-T) for 1 h. After rinsing with TBST, the membranes were incubated with the indicated primary antibodies in 3% skim milk in TBST overnight at 4 °C. Excess primary antibody was discarded by washing the membrane 3 times with TBST. The membranes were then incubated with 0.1 μg/mL of peroxidase-labeled secondary antibody (for rabbits or mice) for 2 h. After three washes with TBST, the bands were treated with ECL Western blotting detection reagent (Thermo Fisher Scientific, Waltham, MA, USA), using an ImageQuant LAS 4000 (GE Healthcare, Chicago, IL, USA). All detected bands were quantified by ImageJ and normalized to the intensity of the loading control protein (β-actin). In the ‘mock’ group, the ratio between the experimental protein and the loading control protein was calculated to be 1×. All normalized proportions were expressed as fold changes compared to the ‘mock’ group.

### 4.14. Statistics

Mean values ± SEM were calculated from the data collected from the three independent experiments performed on separate days. For each mice experiment group, the mean ± SEM were analyzed from the data obtained from the experiments of 5 mice per group and displayed as bar or dot graphs. One-way ANOVA was used to determine the significance (*p* value) and Tukey’s post-hoc test was used after one-way ANOVA. * indicates that differences between the two indicated groups were considered significant at *p* < 0.05.

## 5. Conclusions

Considering the results of this comprehensive study, CDE showed a therapeutic effect by regulating T cell activity through keratin protection from AD, a typical disease caused by immunological imbalance of Th1/Th2, and demonstrated an AD-modulating effect in vitro and vivo. This is a new report on the unknown bioactivity of CDE; therefore, CDE has the potential to become a novel AD treatment.

## Data Availability

Data is contained within the article.
